# Progressive Muscle Weakness and Dysphagia in Late-Onset Systemic Lupus Erythematosus: A Case Report

**DOI:** 10.7759/cureus.79327

**Published:** 2025-02-19

**Authors:** Sho Fukuda, Kasumi Nishikawa, Ryuichi Ohta

**Affiliations:** 1 Fmaily Medicine, Unnan City Hospital, Unnan, JPN; 2 Family Medicine, Unnan City Hospital, Unnan, JPN; 3 Community Care, Unnan City Hospital, Unnan, JPN

**Keywords:** corticosteroid therapy, dysphagia, family medicine, general medicine, late-onset systemic lupus erythematosus, multidisciplinary management, myositis, neuromuscular involvement, rural

## Abstract

Late-onset systemic lupus erythematosus (SLE), defined as SLE developing after age 50, presents distinct clinical features influenced by immunosenescence. Compared to early-onset SLE, it often manifests with nonspecific symptoms such as myositis-like weakness, serositis, and subtle systemic features, complicating timely diagnosis. Given the complexity of comorbidities in elderly patients, distinguishing late-onset SLE from other conditions is critical for appropriate management. We report a case of a 76-year-old man who presented with progressive lower limb weakness and dysphagia. Initial investigations, including laboratory tests, autoantibody profiling, imaging, and nerve conduction studies, led to the diagnosis of late-onset SLE with neuromuscular involvement. The patient exhibited significant systemic symptoms, including muscle weakness, hematologic abnormalities, and pulmonary involvement. Prompt initiation of methylprednisolone pulse therapy followed by intravenous immunoglobulin (IVIG) and oral corticosteroids led to a marked improvement in muscle strength and swallowing function. Within 36 days, he regained independence in activities of daily living (ADL) and was transferred for rehabilitation. This case highlights the diagnostic challenges and therapeutic considerations in late-onset SLE. Myositis and dysphagia, though uncommon, can be prominent in elderly patients, necessitating a high index of suspicion. Early recognition and a multidisciplinary approach are essential for optimizing treatment and functional recovery.

## Introduction

Late-onset systemic lupus erythematosus (SLE), defined as SLE that develops after age 50, exhibits distinct characteristics compared to early-onset cases [1]. It is thought to be influenced by immunosenescence and typically presents with nonspecific symptoms such as arthralgia, serositis, mild rashes, and renal involvement [2]. The diagnosis relies on the ACR/EULAR (American College of Rheumatology/European League Against Rheumatism) 2019 criteria, with a positive antinuclear antibody (ANA) test forming the basis, making the differentiation from other diseases crucial [3]. Treatment options for mild cases include hydroxychloroquine (HCQ), while moderate cases are managed with low-dose corticosteroids, and severe cases may require immunosuppressants or belimumab [3]. In elderly patients, attention must be given to comorbidities and drug interactions to minimize adverse effects [4]. Low-dose corticosteroid therapy is recommended to mitigate the risks of osteoporosis and infections [5]. For late-onset SLE, treatment goals often focus on disease control and improving quality of life (QOL) [5].

Late-onset SLE frequently presents with nonspecific symptoms, making the diagnosis challenging. In elderly patients, multiple comorbidities can obscure clinical manifestations, potentially delaying diagnosis and treatment [6]. We report a case of late-onset SLE that initially presented with difficulty in movement. In this case, differentiation from comorbid conditions posed a significant challenge during the early diagnostic process. However, the patient was ultimately diagnosed with SLE and received appropriate treatment with intravenous methylprednisolone, high-dose intravenous immunoglobulin therapy, and hydroxychloroquine. This report aims to review the patterns of disease onset, diagnostic, and therapeutic processes and highlight the importance of a comprehensive approach by general practitioners in managing elderly patients, contributing to practical solutions for similar cases.

## Case presentation

A 76-year-old man presented to a regional hospital with difficulty moving his body. He had been experiencing general fatigue for several months. One week before his visit, he gradually began to lose strength in both lower limbs, which made daily activities challenging. On the day of admission, he was unable to move due to severe weakness in both lower limbs and was transported to the hospital by ambulance. He had no preceding cold symptoms, no known contact with infected individuals, no history of outdoor exposure, no pets, and no recent contact with animals or insect bites. Routine health checkups, including upper and lower gastrointestinal endoscopies performed earlier that year, showed no significant abnormalities. The patient had been independent in activities of daily living (ADL), smoked 12-13 cigarettes per day, and did not consume alcohol. His medical history included cerebral infarction, chronic obstructive pulmonary disease (COPD), hypertension, dyslipidemia, and an abdominal aortic aneurysm. He was on 5 mg of amlodipine.

**Figure 1:**
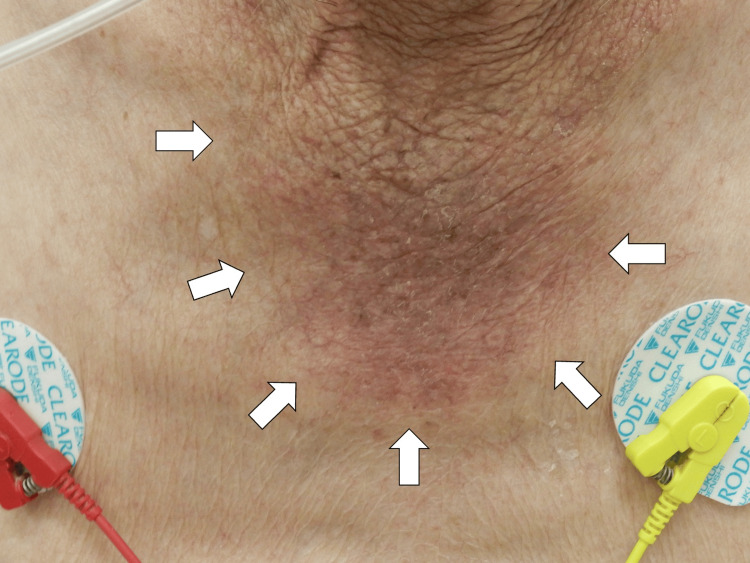
Scraping-like eczema extending from the neck to the chest (white arrows)

On arrival, his vital signs were as follows: conscious and alert, body temperature 35.6°C, blood pressure 161/77 mmHg, pulse rate 56 bpm, SpO₂ 96% (on room air), and respiratory rate 20/min. Physical examination revealed proximal muscle weakness in all extremities, with muscle strength rated 4/5 on the Medical Research Council scale for both upper limbs and 4/5 for the iliopsoas and gastrocnemius muscles in both lower limbs. Decreased deep tendon reflexes were observed in all extremities, and the vibratory sensation was markedly reduced in both lower limbs. Skin examination revealed a scraping-like eczema extending from the neck to the chest (Figure 1).

A cardiac exam detected a systolic ejection murmur without radiation to the neck, and late crackles were heard at the bases of both lungs. There was no warmth, swelling, or tenderness in the joints, but grasping pain was noted in both thighs. Laboratory tests revealed elevated C-reactive protein (CRP), creatine kinase (CK), anemia, leukopenia, thrombocytopenia, and decreased complement levels (Table 1).

**Table 1:** Initial laboratory data of the patient CRP, C-reactive protein; Ig, immunoglobulin

Parameter	Level	Reference
White blood cells	3.2	3.5–9.1 × 10^3^/μL
Neutrophils	63.4	44.0–72.0%
Lymphocytes	13.9	18.0–59.0%
Hemoglobin	11.7	11.3–15.2 g/dL
Hematocrit	34.5	33.4–44.9%
Mean corpuscular volume	95.6	79.0–100.0 fl
Platelets	2.5	13.0–36.9 × 10^4^/μL
Total protein	6.9	6.5–8.3 g/dL
Albumin	2.2	3.8–5.3 g/dL
Total bilirubin	0.4	0.2–1.2 mg/dL
Aspartate aminotransferase	75	8–38 IU/L
Alanine aminotransferase	51	4–43 IU/L
Lactate dehydrogenase	420	121–245 U/L
Blood urea nitrogen	20.2	8–20 mg/dL
Creatinine	0.64	0.40–1.10 mg/dL
Serum Na	137	135–150 mEq/L
Serum K	4.0	3.5–5.3 mEq/L
Serum Cl	103	98–110 mEq/L
Ferritin	692.8	14.4–303.7 ng/mL
CRP	2.46	<0.30 mg/dL
IgG	3240	870–1700 mg/dL
IgM	204	35–220 mg/dL
IgA	967	110–410 mg/dL
Complement 3	24	86-160 mg/dL
Complement 4	2	17-45 mg/dL
Urine test	-	-
Leukocyte	Negative	Negative
Protein	1+	Negative
Blood	1+	Negative

Polyclonal hypergammaglobulinemia was also observed. Urinalysis showed proteinuria and hematuria.

**Figure 2:**
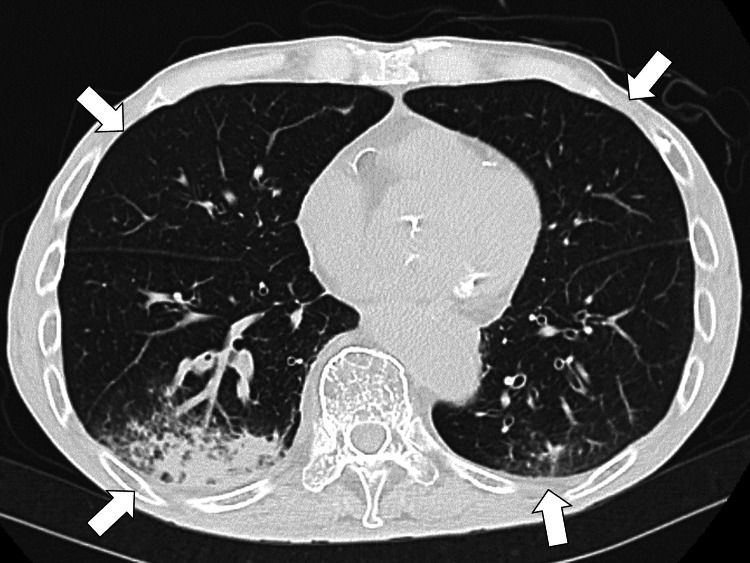
Contrast-enhanced computed tomography (CT) revealing scattered ground-glass opacities and infiltrations in both lungs (white arrows)

Contrast-enhanced computed tomography (CT) of the neck to pelvis, performed to investigate suspected malignant lymphoma or deep-seated abscess, revealed scattered ground-glass opacities and infiltrations in both lungs (Figure 2).

**Figure 3:**
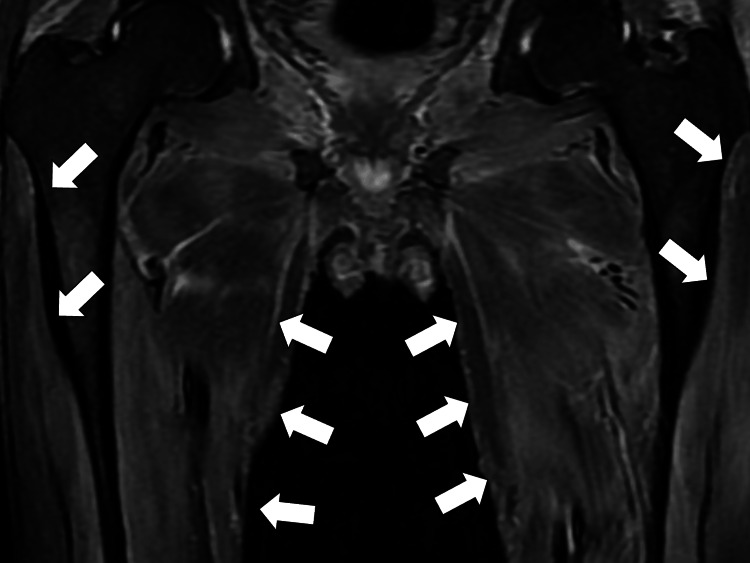
Magnetic resonance imaging (MRI) of both thighs showing high-signal areas in the muscles on short tau inversion recovery (STIR) imaging (white arrows)

Magnetic resonance imaging (MRI) of both thighs, conducted to evaluate the cause of lower limb pain, showed high-signal areas in the muscles on short tau inversion recovery (STIR) imaging (Figure 3).

The transthoracic cardiac ultrasound did not show any vegetation. On the second day of hospitalization, excessive salivation was observed, and an otolaryngology consultation revealed moderate laryngeal muscle weakness and dysphagia. On the third day, results from outsourced blood tests showed positive ANA at a titer of 1:640 and elevated anti-Sm antibodies at 10.8 U/mL (normal range, less than 7). On the fourth day, nerve conduction studies showed delayed conduction velocities.

Based on these findings and ACR/EULAR 2019 criteria, the patient was clinically diagnosed with SLE [3]. Suspecting aspiration pneumonia due to the presence of a wet cough and purulent sputum, intravenous ceftriaxone (2 g/day) was initiated on the first day. For the treatment of SLE with complications including pneumonitis, bone marrow involvement, and peripheral neuropathy, intravenous methylprednisolone (1,000 mg/day) was administered for three days starting on the sixth day. This was followed by five days of high-dose intravenous immunoglobulin therapy (0.4 g/kg/day) and the initiation of oral hydroxychloroquine (200 mg/day) and prednisolone (30 mg/day) on the ninth day.

The patient's swallowing function markedly improved, allowing oral intake by the 14th day of the admission. Simultaneously, muscle strength in all extremities improved, and by the 24th day, the patient transitioned from being bedridden to regaining independence around the bed. By the 30th day, blood cell counts and complement levels had normalized. Prednisolone was tapered at a rate of 5 mg/week. The patient had a favorable clinical course and was transferred to a rehabilitation ward on the 36th day of the admission.

## Discussion

Through this case, we highlight that late-onset SLE can present with myositis-like symptoms. This may lead to swallowing dysfunction, posing a significant risk of rapid functional decline in ADL. Despite the patient's advanced age, this case underscores the importance of promptly investigating systemic symptoms and initiating appropriate therapies, including corticosteroids and immunoglobulin therapy, to substantially improve systemic manifestations and restore the potential for independent living. Referencing prior evidence, we delve into the clinical and pathophysiological aspects of late-onset SLE, focusing on the challenges in diagnosis, the importance of tailored treatment strategies, and broader implications for management in elderly patients.

Late-onset SLE presents atypical symptoms, including nonspecific musculoskeletal complaints and subtle systemic features, making diagnosis challenging in older populations. Evidence suggests that elderly patients are more likely to exhibit mild skin involvement and less renal disease than younger individuals, although neuropsychiatric and musculoskeletal symptoms may be prominent [7,8]. In our case, the patient presented with proximal muscle weakness and dysphagia, both of which are less common but documented in SLE-related myositis [9,10]. The presence of these symptoms, combined with a history of multiple comorbidities, required a high index of suspicion and comprehensive evaluation using autoantibody profiles and imaging studies.

The pathophysiology of late-onset SLE may differ due to age-related changes in the immune system, such as immunosenescence, which leads to a predisposition to autoimmunity and a different spectrum of organ involvement [11]. Myositis and neuromuscular involvement, as seen in this patient, are rare but have been reported in late-onset SLE and can manifest as a result of immune-mediated inflammatory processes [12]. Dysphagia in such cases is attributed to inflammatory myopathy affecting the pharyngeal and esophageal muscles, highlighting the need for vigilance in detecting subtle signs of neuromuscular dysfunction. Dysphagia can critically affect mortality and the possibility of discharge to their home because of the care burden in their home, especially in rural contexts, as in this case [13,14]. Thus, prompt detection and interventions for dysphagia in systemic inflammatory diseases are critical.

Treatment goals in late-onset SLE focus on controlling disease activity while minimizing adverse effects, particularly in elderly patients with comorbidities. Corticosteroids remain the cornerstone of treatment, and prior studies have demonstrated their efficacy in rapidly controlling inflammation and improving systemic symptoms in severe SLE cases [15]. In this case, the patient’s rapid improvement following high-dose methylprednisolone and intravenous immunoglobulin (IVIG) aligns with evidence supporting the use of these therapies for refractory or severe manifestations, such as myositis and bone marrow suppression [16]. Hydroxychloroquine has been shown to reduce disease flares and improve long-term outcomes, reinforcing its use as maintenance therapy in this patient [17]. Hydroxychloroquine can control the type 1 interferon pathway, triggering inflammation in SLE [17]. In the management of the critical symptoms of SLE, intensive treatments should be implemented promptly, such as steroid pulse and IVIG. Still, hydroxychloroquine should be used based on the pathophysiology of SLE to control excessive activation of the type 1 interferon pathway.

This case emphasizes the importance of early recognition and a multidisciplinary approach in managing late-onset SLE. General physicians and specialists must collaborate to ensure timely diagnosis and tailored treatment strategies, particularly in elderly patients with complex clinical presentations of autoimmune diseases [18]. As highlighted in this case, rehabilitation support is critical for restoring ADL and improving quality of life. Previous evidence has underscored the role of a multidisciplinary approach in improving outcomes for elderly patients by addressing both medical and functional aspects of care in rural contexts [19,20]. As this article shows, older patients with acute exacerbation of autoimmune diseases should be managed promptly through interprofessional collaboration in rural contexts.

## Conclusions

Late-onset SLE can present with severe and rapidly progressive systemic symptoms, including myositis and dysphagia, which significantly impair ADL and quality of life. However, referencing prior evidence, we demonstrate that timely and appropriate interventions, such as corticosteroids and immunoglobulin therapy, can improve systemic symptoms and functional outcomes. This case highlights the importance of individualized treatment strategies and a comprehensive, multidisciplinary approach in the management of late-onset SLE in elderly patients.
